# A Pilot Study of CK19, CK20 and GCC mRNA in the Peripheral Blood as a Colorectal Cancer Biomarker Panel

**Published:** 2016

**Authors:** Pouria Mohammadi, Massoud Saidijam, Arastoo Kaki, Katayoon Etemadi, Nooshin Shabab, Reza Yadegarazari

**Affiliations:** 1 *Research Center for Molecular Medicine, Hamadan University of Medical Sciences, Hamadan, Iran.*; 2 *Department of Genetic & Molecular Medicine, Hamadan University of Medical Sciences, Hamadan , Iran.*

**Keywords:** Colorectal cancer, cytokeratin 19, cytokeratin 20, guanylyl cyclase C, biomarker

## Abstract

Colorectal cancer remains one of the major cancer- related deaths despite progress in the treatment during past decades. Detection of disease at earlier stages reduces its mortality. The aim of current study was to investigate expression of Cytokeratin 19 (CK19), Cytokeratin 20 (CK20) and Guanylyl Cyclase C (GCC) mRNA in peripheral blood of non- metastatic colorectal cancer patients which may result into introducing of an early detection test. 25 patients with colorectal cancer and 25 healthy controls were recruited. Blood was obtained from all individuals. Expression of CK19 and CK20 and GCC mRNA and 18SrRNA (as reference gene) were determined based on real- time RT-PCR on total RNA from blood. CK19, CK20 and GCC expression had been detected in 68%, 76% & 52% of patient group, respectively, which was higher than healthy group, with 8%, 32% and 0% expression, respectively (p<0.05). CK20 was over-expressed 8- fold more in patients compared to controls. Similar result was found for CK19 with 4- fold over- expression. Sensitivity and specificity of combination of markers were 88% and 68%, respectively. Current data suggest that the detection of CK20 & CK19 as relative sensitive markers may become a valuable tool for primary diagnosis of colorectal cancer in early stages. GCC could be considered as a specific tumor marker for detection of colorectal cancer. Higher expression of these markers in patients may be considered as a relative good tool for the diagnosis of disease in non- metastatic stages.

Despite significant progress in decreasing mortality and improving survival rates in patients, cancer is still the major cause of death worldwide (1). Colorectal cancer is the third most commonly diagnosed cancer in males and the second in females (2). During the last decades, considerable progress has been achieved towards improving survival of patients. Since more than 60% of colorectal cancer has been identified at the symptomatic phases with lower rate of long-term survival, diagnosis of disease at the earlier asymptomatic stages and effective screening is critical (3). In addition, introducing the new therapeutic agents and the discovery of predictive and prognostic biomarkers will enable oncologists to individualize therapeutic strategies by increasing drug effectiveness and decreasing incompatible side effects in colorectal cancer patients (4, 5).

The use of Real-time-PCR to analyze the blood of cancer patients for the detection of mRNA expressed in tumor cells is one of the interesting concepts in the management of cancer (6). Many mRNA markers of colorectal cancer have been studied in the peripheral blood of the patients (7). By evaluating mRNA expression of specific tumor markers, Real– time PCR can detect cancer cells in peripheral blood of cancer patients compared with healthy groups because the tumor cells, shed from the primary tumor mass into the blood stream, could be tracked in the early stages of the disease (8).

A number of tumor markers, such as cytokeratin19 and cytokeratin 20 and guanylyl cyclase C have been shown to be specifically and stably expressed in primary and metastatic colorectal cancer cells and, as such, have been used for the efficient detection of circulating tumor cells in peripheral blood (9, 10). Cytokeratin is one of the intermediate filaments, which is mainly found in epithelial cells and particularly useful tools in oncology diagnostics. Cytokeratin-based tumor marker assays may be considered as simple, noninvasive, cheap, and reliable predictive tests and offer a tool for more efficient management before conventional methods (11).

Cytokeratin 20 (CK20) is a low molecular weight member of the Cytokeratin family of proteins that is expressed in primary colorectal tumors and their metastases (12). Cytokeratin19 (CK19), because of restricted range of its expression, has been considered as a tool for detecting and identifying cancer cells in the peripheral blood by PCR analysis. It has previously been shown to serve as circulating tumor cells associated marker in colorectal cancer patients and has been used to detect disseminated tumor cells and occult metastases in the patients. So, CK19 could improve the early detection of distant organmetastases (13, 14).

Guanylyl cyclase C (GCC), found as the target for heat-stable enterotoxin of *Escherichia coli* is one of studied biomarkers of colorectal cancer (15). GCC signaling has been shown in normal intestinal and colorectal carcinoma (CRC) cells. Expression of GCC in circulating tumor cells of CRC patients was detected by PCR, but normal subjects and nonmalignant intestinal pathologies had shown negative results. GCC is mainly studied in diagnosis, staging and management of colorectal cancer metastases (16, 17).

However, since some of the markers used to detect circulating tumor cells are not cell type-specific, recent studies have suggested that the assessment of a combination of circulating tumor cells markers could increase the efficiency of circulating tumor cell detection, as compared to the analysis of single markers (7). In this study, we aimed to investigate expression of CK19 and CK20 and GCC mRNA in circulating tumor cell in peripheral blood of colorectal cancer patierts without distant metastases by Real-time PCR assay.

## Materials and methods

**Study groups**

We recruited 25 patients (12 males, 13 females) with CRC from stages I, II and III of disease (7, 10 and 8 patients, respectively) who underwent surgical treatment at the Gastrointestinal Department of Beheshti Hospital, Hamadan , & General Surgery Department of Imam Khomeini Hospital, Tehran. Routine pathological examina-tions were used to confirm the diagnosis of disease. Location of tumor was colon (16 patients, 64%) and rectum (9 patients, 36%). Patients with a known second neoplastic disease or history of curative surgery, chemotherapy and/or radiotherapy before blood sampling were excluded from the study. 25 healthy volunteers (13 males, 12 females) referred to colonoscopy unit of Beheshti Hospital of Hemadan, with normal results of pathological examinations, were also included. All study protocols were approved by the Local Ethics Committee of Hamadan University of Medical Sciences and informed consent was obtained from all study participants.

**Blood sampling**

After a brief explanation of study purpose and obtaining informed consent, 10 ml peripheral venous blood was obtained and collected in sodium EDTA containing tubes, kept on ice, transferred to laboratory and processed within 1 hour after collection.

**RNA extraction**

40 ml of erythrocyte lysis buffer was added to 10 ml blood sample to eliminate RBCs. The buffer composed of 0.32 M sucrose, 10mM Tris- HCL pH 7.5, 5mM MgCl2 and 1% Triton X-100. The mixture was held on ice for 30 min before centrifugation. Precipitated WBC pellet was washed by PBS solution. Second steps of lysis (with 20 ml of the buffer) and wash was done for complete elimination of hemoglobin.

RNA extraction was performed on WBC pellet (containing probable tumor cells) using the RNeasy Midi Kit (Qiagen, Hilden, Germany). The entire isolated RNA was dissolved in 300 μl RNase-free water. Integrity of RNA was confirmed by 1% agarose gel-electrophoresis. Optical density (OD) measurement via Nano- Drop (BioTech, USA) was also used for the assessment of RNA purity which was ascertained by an OD260/280 ratio= 1.8- 2. RNA concentration wasdetected in range of 0.6- 2.3 μg/ μl.

**Reverse transcription**

One µg of extracted total RNA underwent reverse transcription. QuantiTect Reverse Transcription Kit (Qiagen, Hilden, Germany) was used for reverse transcription. Integrity of produced cDNA was confirmed by 2.5% agarose gel-electrophoresis. Reference gene expression was used for assessment of template cDNA quality. Based on similar study in our center, 18S rRNA was chosen as internal control reference gene (7). Primers design was done by Allele ID 7 software (PREMIER BIOSOFT). Primers properties are shown in Table 1. Primers efficacy had been checked by preliminary tests on positive and negative controls. Primers efficiencies were calculated.

**Real-time qRT-PCR assay**

Real- time qRT-PCR assays for the determination of CK19 and 20 and GCC mRNAs were constructed using the QuantiTect® SYBR® Green PCR Kit (Qiagen, Hilden, Germany) in CFX96 Real-time PCR detection system (BioRad, USA). Positive and negative controls were used for quality control of the process. Each reaction was conducted in 50 μl final volume containing 25 μl 2x QuantiTect SYBR Green PCR Master Mix, 2 μl template cDNA and 10 pmole of each forward & reverse primers.

**Table1:** Properties and amounts of primers used in real-time RT-PCR assays of marker and reference genes

**mRNA** ** Marker**	**Accession number**	**Forward primer** **5' ** **→** ** 3'**	**Reverse primer** **5' ** **→** ** 3'**	**Amplicon length (bp)**
*GCC*	NM-004963	GGGTGGCTGTCCTTTAGTT	GTAGCGTTCACAGTCACA	191
*CK19*	NM-002276	TCCGAACCAAGTTTGAGAC	AATCCACCTCCACACTGA	222
*CK20*	NM-019010	ACGCCAGAACAACGAATACC	TTCAGATGACACGACCTTGC	208
*18S rRNA*	X03205	GTAACCCGTTGAACCCCATT	CCATCCAATCGGTAGTAGCG	151

*GCC*: Guanylyl Cyclase C

*CK19*: Cytokeratin 19

*CK20*: Cytokeratin 20

*18S rRNA*: 18S ribosomal RNA

Real- time PCR program was performed by an initial denaturation at 95 ºC for 5 min, followed by 40 cycles of denaturation at 94 ºC for 15 s, annealing at 55 ºC for 30 s and extension at 72ºC for 30 s, followed by data acquisition step. Melting curve analysis was used for the assessment of true amplification of markers and reference gene. For the purpose, melting temperature of specific products was considered for discrimination of false and true amplification curves.

**Statistical analysis**

The sample size had been calculated based on the difference between proportions of positive ratios in two groups, reported in previous similar studies (13, 14). All statistics were calculated using the SPSS software (Version 10). T-Test was applied for comparison of two means. Two- sample binomial test was used for comparisons of the prevalence between study groups. A p-value < 0.05 was considered significant. ΔΔCt method was applied for the estimation of difference level of gene expression between studied groups.

## Results

The mean age of patient and healthy groups was 61 years (range: 16 – 87 years) and 62.3 years (range: 25–88 years), respectively. There was no significant difference between patient and healthy groups in mean of age (P= 0.80).

**Primer efficiency and expression levels of reference gene**

Ct value of 18S rRNA was determined in samples to assay the reference gene expression in patient and healthy groups. The calculated 18S rRNA Ct values were 22.7± 2.9 in patient and 21.8± 4.8 in healthy groups. There was no significant statistical difference between the two study groups for *18S rRNA* gene expression (P= 0.45).The results were similar between men and women (P= 0.16) in both main groups of the study. Therefore, this marker could be considered as a reference for the normalization of our biomarkers expression in blood samples.

Efficiency of reactions was 94%, 97%, 101% and 92% for 18S rRNA, CK19, CK20 and GCC mRNA, respectively.

**Expression rate of markers in patients and healthy volunteers**

Positive rate of markers in patients and healthy volunteers was calculated. According to the findings, sensitivity and specificity as well as false positive and false negative rates were revealed for each marker.

17 out of 25 patients were positive for CK19 mRNA and 2 subjects showed marker expression in control group. For CK20 mRNA, 19 patients were positive and 8 persons in control group showed positive results. GCC mRNA was detected in 13 patients and none of 25 persons showed marker expression in control group. Combined markers analysis showed that at leastone of the 3 makers was positive in 22 patients and 8 healthy persons. Therefore, sensitivity of markers was calculated 22 out of 25 (88%) and specificity was determined 17 out of 25 (68%) (Table 2).

**Table 2:** Calculated statistical parameters of CK19/20 and GCC mRNA in peripheral blood of 25 colorectal cancer patients and 25 healthy volunteers

**Marker**	**Sensitivity**	**Specificity**
GCC	52%	100%
CK19	68%	92%
CK20	76%	68%
GCC+ CK19+ CK20	88%	68%

GCC: Guanylyl Cyclase C

CK19: Cytokeratin 19

CK20: Cytokeratin 20

Combination markers sensitivity (88%) was higher than GCC mRNA sensitivity (52%) (P= 0.003) and CK19 mRNA sensitivity (68%) (P= 0.04) but did not have meaningful difference with CK20 mRNA sensitivity (76%) (P= 0.13).

Comparison of single and marker combined with positive ratios between male and female patients, between colon and rectal cancer patients and between different stages of disease did not show meaningful difference.

**Expression levels for the markers between patient and healthy volunteers**

To assay the expression levels of markers in patients and healthy groups, we calculated ΔCt of both CK19 and CK20 using the following formula: [Ct value of maker- Ct value of reference] and then, the mean of this value was calculated in two study groups.

For CK19, the mean of ΔCt were 7.73 in patient group and 9.69 in healthy group. These parameters were 7.24 and 10.23 for CK20, respectively. Then, we calculated ΔΔCt of CK19 and CK20 using the following calculation: [ΔCt of patient group - ΔCt of healthy group]. The parameter was -1.96 for CK19 and -2.99 for CK20.

**Fig. 1:**
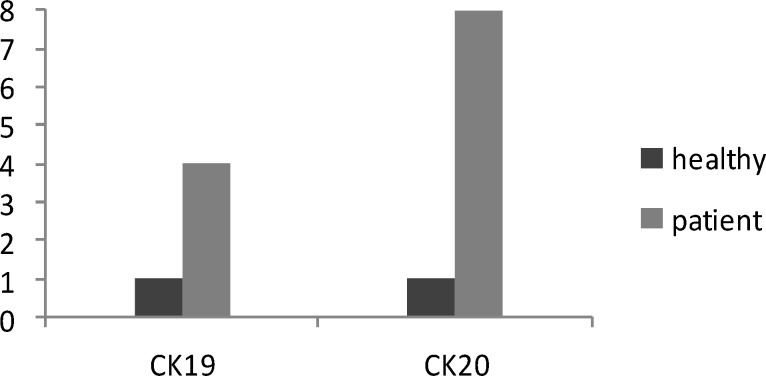
Comparison of calculated expression levels for the Cytokeratin19 (*CK19*) and Cytokeratin20 (*CK20*) in peripheral blood of 25 colorectal cancer patients and 25 healthy volunteers. Numbers in Y axis indicate relative expression levels

According to Livak method, which explains that 2 ^-ΔΔCt^ value should be considered as indicator of difference between the expression level of two groups, expression level of *CK19* in patients was approximately calculated 4 (2^1.96^) times higher than healthy volunteers. For *CK20*, the expression level was 8 (2^3.99^) times higher in patient group. None of the subjects in control group showed *GCC* expression, so we did not include GCC in this part (Fig.1).

## Discussion

The aim of the present pilot study was to find blood biomarkers for the early diagnosis of CRC. Biomarkers including mRNA markers that have appropriate features and even small amount of them can be found with Real- time RT-PCR in tissues and bloodstream, are useful tools for early detection of CRC (18). A few mRNA biomarkers have been studied as peripheral blood marker for the early detection of CRC (19). Accordingly, in this research, we evaluated GCC, CK19 and CK20 mRNA in the peripheral blood of patients and healthy groups.

An important source of blood mRNA biomarkers in cancer, is circulating cancer cells (CTC) (8). The amount of biomarkers in the peripheral blood is low, especially in earlier stages of disease and thus, multiple blood sampling methods and circulating tumor cell enrichment techniques or consecutive tests on each sample is needed for increasing the sensitivity of the test (20). Concurrent assay of multiple markers is another solution for the problem (21). In the present study, sensitivity of multiple marker analysis was meaningfully higher than GCC and CK19 marker sensitivity. However, this comparison was not meaningfully notable for CK20. The results indicate that combination of markers increase the sensi-tivity of the diagnostic test in comparison with single low-sensitive markers. Similar result has been reported by Khair G et al. (21). Combina-tion analysis of these markers in total and cell fractions of blood in patients with solid tumors in stages III–IV showed the same data (22). However, paradoxi-cal results have been found by Bustin et al. (23).

The specificity and false positive rate were also evaluated in this study. In the case of cancer, the false positive is very important because it may result into mental health problems due to cancer misdiagnosis and wasting time and money for healthy individuals. Thus, enhancing the specificity and minimizing the false positive rate is very critical (24). False positive results of tumor marker expression in healthy group may be due to illegitimate transcription or background expression and lead to decrease in specificity of the test (5). To increase the specificity of markers, designing a cut-off point strategy may be useful. In this study, we found that cytokeratin markers had shown false positive results. Then, we calculated the difference between gene expression rates of *CK19* and *CK20* in two groups. Their higher expression in patient group could be considered as an indicator for offering a cut-off value for the discrimination of true and false positive results. However, we could not report this value because of low sample size as well as non- quantitive method of our study.

In this study, the specificity of GCC was estimated 100%. GCC mRNA expression has been detected in all stages of primary and metastatic CRC and any grade and anatomic location of tumor, but only few studies evaluated peripheral blood GCC expression in CRC patients (25, 26). According to many studies, *GCC* could be used as a rather specific biomarker for diagnosis, staging of disease and evaluation of treatment efficacy as well as prognosis interpretation of CRC. Sensitivity and specificity of GCC mRNA have been reported in a wide range in different investigations (9, 25, 26).

Colonoscopy, as gold standard test for CRC diagnosis, has 95% sensitivity and 100% specificity which is higher than the other routine screening procedures of colorectal cancer (27, 28). By comparison with colonoscopy, our primary findings showed relatively optimistic results. Therefore, it may be assumed as a tool for detection of non-metastatic disease in people with no tendency to invasive screening procedures. However, it should not be considered as a replacement method for colonoscopy.

In conclusion, the results of this study suggest that mRNA markers of peripheral blood may be considered as useful tools to find non-metastatic CRC by real-time RT-PCR. Combination of sensi-tive and specific makers as a panel of diagnostic test and considering cut-off value strategy increase the total sensitivity and specificity of the panel as a primary non-invasive test for the diagnosis of CRC in non- metastatic stages. However, more widesp-read studies are required to confirm our findings.
